# BIOCAT: a pattern recognition platform for customizable biological image classification and annotation

**DOI:** 10.1186/1471-2105-14-291

**Published:** 2013-10-04

**Authors:** Jie Zhou, Santosh Lamichhane, Gabriella Sterne, Bing Ye, Hanchuan Peng

**Affiliations:** 1 Department of Computer Science, Northern Illinois University, DeKalb, IL 60115, USA; 2 Life Sciences Institute and Department of Cell and Developmental Biology, University of Michigan, Ann Arbor, MI 48109, USA; 3 Allen Institute for Brain Science, Seattle, WA 98103, USA

## Abstract

**Background:**

Pattern recognition algorithms are useful in bioimage informatics applications such as quantifying cellular and subcellular objects, annotating gene expressions, and classifying phenotypes. To provide effective and efficient image classification and annotation for the ever-increasing microscopic images, it is desirable to have tools that can combine and compare various algorithms, and build customizable solution for different biological problems. However, current tools often offer a limited solution in generating user-friendly and extensible tools for annotating higher dimensional images that correspond to multiple complicated categories.

**Results:**

We develop the BIOimage Classification and Annotation Tool (BIOCAT). It is able to apply pattern recognition algorithms to two- and three-dimensional biological image sets as well as regions of interest (ROIs) in individual images for automatic classification and annotation. We also propose a 3D anisotropic wavelet feature extractor for extracting textural features from 3D images with xy-z resolution disparity. The extractor is one of the about 20 built-in algorithms of feature extractors, selectors and classifiers in BIOCAT. The algorithms are modularized so that they can be “chained” in a customizable way to form adaptive solution for various problems, and the plugin-based extensibility gives the tool an open architecture to incorporate future algorithms. We have applied BIOCAT to classification and annotation of images and ROIs of different properties with applications in cell biology and neuroscience.

**Conclusions:**

BIOCAT provides a user-friendly, portable platform for pattern recognition based biological image classification of two- and three- dimensional images and ROIs. We show, via diverse case studies, that different algorithms and their combinations have different suitability for various problems. The customizability of BIOCAT is thus expected to be useful for providing effective and efficient solutions for a variety of biological problems involving image classification and annotation. We also demonstrate the effectiveness of 3D anisotropic wavelet in classifying both 3D image sets and ROIs.

## Background

Advances in biological imaging in the past decade [1-3] have brought the field of bioimage informatics to a new scale [4,5]. Multi-dimensional microscopic images have played significant roles in biology discovery, such as exploring neuron system’s structure and function during neuronal development under genetic manipulation [6]. Much effort has been spent on various aspects of informatics such as storing, visualizing and analyzing high dimensional and content-rich biological images [5]. Such efforts have yielded programs like ImageJ [7], Vaa3D [8], Cell Profiler [9], FARSIGHT [10], Icy [11], OME [12] and BISQUE [13].

Pattern recognition algorithms have also gained momentum in automatic analysis and quantification of biological images. Pattern recognition uses a trained classifier to automatically assign an image to a category of interest. To build the trained classifier, the images are typically transformed into a feature vector via feature extraction and possibly followed by a subsequent selection [14]. The trained model can then be used to predict unseen images’ category, with applications such as protein expression annotation and characterization, cell phenotype determination/counting, and subcellular protein arrangement [15-20].

Several pattern recognition-based tools for biological image classifications are available. Details of the commonly known free tools are compared in Table 1. Table 1 shows that current tools have their various limitations. For example, almost all the related tools use a fixed pattern recognition model (one fixed classifier and an often fixed set of features). Some of them only work with 2D images (e.g. Wndchrm [21] and Cell Profiler [9]) or require commercially licensed software. To summarize, several challenges in the field remain to be addressed:

•Adaptability to different problems. In pattern recognition, it is widely believed that no model can work universally well for every problem [23]. A “model” means image feature descriptors, classifiers and the combination of them. Currently most of the bioimage recognition tools provide fixed (or very limited) choice for models.

•Image-oriented multi-dimensional machine learning for bioimage quantification. Platforms for 3D and higher dimensional images classification and analysis lag behind greatly, compared with their 2D counterparts. Machine learning libraries that are not image-oriented, such as Weka [24], fall short on efficiency when classifying high-content biological images.

•Automatic annotation of images, which can be formulated as image classification problems by treating annotation labels as classification targets, are seldom addressed by existing tools other than some efforts for annotating specific images sets [16,25,26].

•Usability for non-technical users. Existing tools often fall short of good usability for biologists who do not have a lot of knowledge about pattern recognition and machine learning algorithms—yet they are the major users of such tools. This has been recognized as a common issue, and usability has recently been suggested as a more highly valued goal in bioimage informatics [27].

**Table 1:** Comparison of existing pattern recognition based bioimage classification tools with BIOCAT (as the writing of the paper)

	**Graphic user interface**	**3D image**	**Classifier**	**Extensible algorithm plugin**	**ROI**	**Automatic comparison among algorithms**	**Required commercial software**	**Platform**
Wndchrm [21]	No	No	Nearest Neighbor	No	No	No	None	Linux, MacOS, Windows
CellProfiler [9]	Yes	No	Boosting	No	Yes	No	Matlab or None	Linux, MacOS, Windows
ImageJ/Fiji (http://rsb.info.nih.gov/ij/)	Yes	Yes	No	Yes	Yes	No	None	All
PSLID/SLIC (http://lanec1web1.compbio.cs.cmu.edu/release/)	No	Yes	BPNet, SVM	No	Yes	No	Matlab	Linux
Ilastik [22]	Yes	Yes	Random Forest	No	Yes	No	None	Linux, MacOS, Windows
**BIOCAT** (http://faculty.cs.niu.edu/~zhou/tool/biocat/)	**Yes**	**Yes**	**Multiple Choices**	**Yes**	**Yes**	**Yes**	**None**	**All**

BIOCAT (BIO-image Classification and Annotation Tool) is developed as an effort to address the above challenges. As shown in the last row of Table 1, it provides a free, open, portable, GUI-based, extensible platform for multi-dimensional bioimage classification and annotation.

Compared with existing tools, it has the advantages of not requiring any commercial licensed software, working with both 2D and 3D images as well as regions of interest (ROI), and the capability to extend with new algorithms. More importantly, it addresses the adaptability challenge by providing a customizable Lego-like solution. The user can interactively build many algorithm chains, each of which consists of a sequence of linked algorithm modules and represents a model for image classification/annotation. These chains can then be compared by BIOCAT, which outputs the most suitable model for the given data. In contrast to other tools that use fixed algorithms, BIOCAT provides a systematic approach for comparing algorithms and their combinations, enables efficient model selection for biological image classification and detection, and thus provides a comprehensive tool for building the effective model for a new task at hand.

The entry for ImageJ in Table 1 is included due to its common use of ROI annotation. ImageJ is more for image processing instead of pattern recognition because of its lack of classifiers except some plugins (a general definition of pattern recognition includes unsupervised algorithms such as clustering. Here we focus on the supervised discriminative models that use trained classifiers). If excluding ImageJ, BIOCAT is the only tool in Table 1 that provides an extensible design allowing new algorithms dynamically loaded as plugins, as far as we know. On the other hand, ImageJ’s processing capabilities such as denoising and enhancement can be used to pre-process the images before they are applied to pattern recognition tasks. The inter-operability of BIOCAT with other tools such as ImageJ will be further discussed in later sections.

The paper is organized as follows: We will describe the design of the tool in Implementation Section. Experiments and discussions are in Experiments and results Section and Discussion Section, followed by conclusions.

## Methods

Pattern recognition algorithms suitable for multiple-dimensional biological image classification and annotation are central components of BIOCAT, which include multiple feature extracting and selection algorithms, as well as classifiers. In this section, we will start with a brief summary of the adaptive design of BIOCAT that allows algorithm modules to work together and form a customizable solution for a given problem. We will then describe the representative algorithm modules (extractors, selectors and classifiers). We will also detail a 3D feature extractor.

### Design of BIOCAT

**Figure 1:**
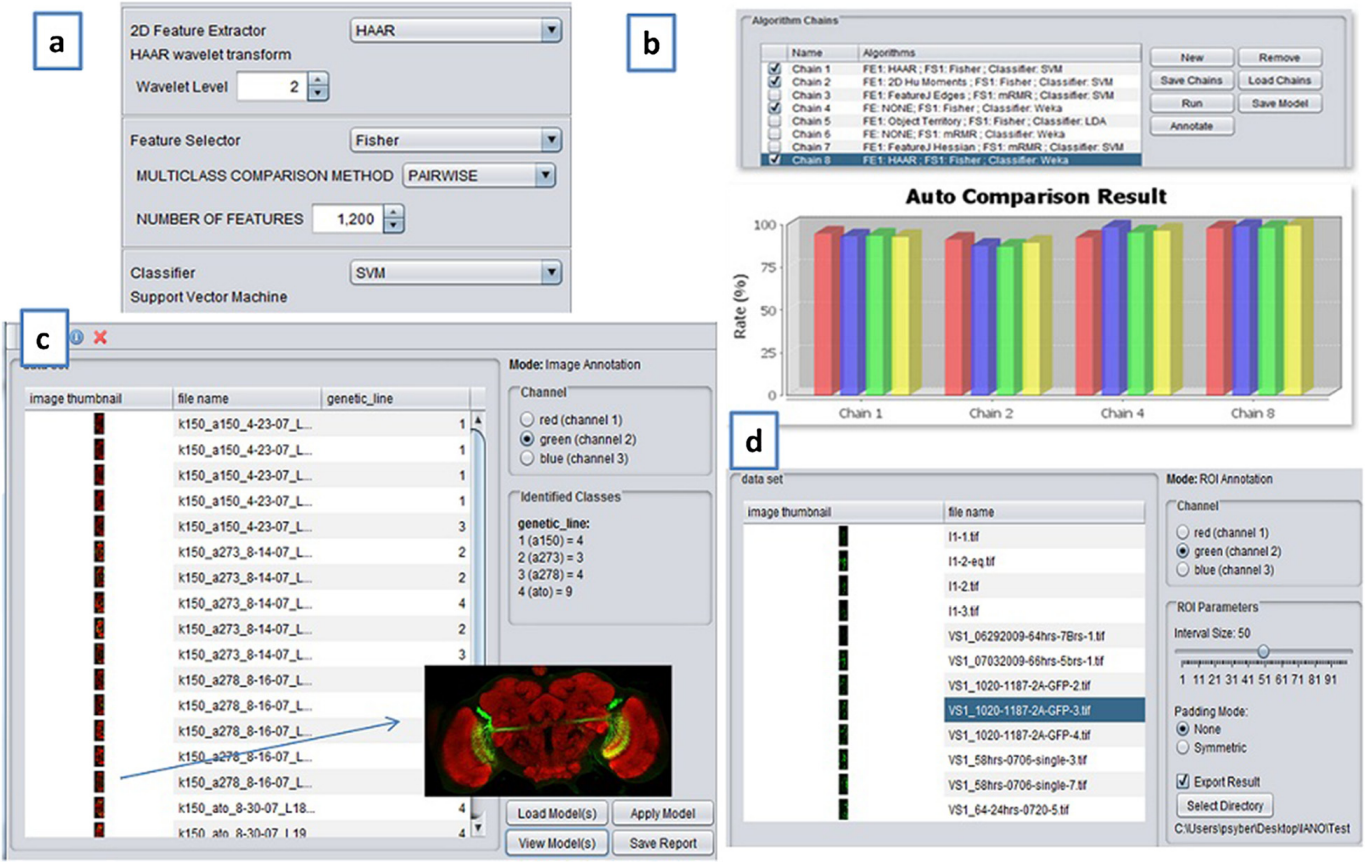
**BIOCAT screenshots. (a)** Algorithm selection panel; **(b)** Algorithm chain list and comparison; **(c)** Image classification/annotation screen and an example image of a neuron population in the fruit fly brain; **(d)** ROI annotation screen.

BIOCAT is designed with 1) usability, 2) extensibility, 3) inter-operability, and 4) portability. Figure 1 shows some screenshots of the BIOCAT GUI.

#### Usability

BIOCAT is intended to be mainly used by biologists with no much experience in pattern recognition (although technical users might also find it useful for algorithm evaluation). While some training is required, we expect that the end users can adaptively build a model that is best fit for their classification problem at hand, facilitated by the GUI of the tool.

As shown in Figure 1, BIOCAT has a user-friendly GUI that permits users to conduct: a) customizable model building, which adaptively selects a model for the given biological image classification/annotation task. The user can do so using a training set and a testing set, or cross validation on a given set. The model can be built, compared and saved. One important feature of BIOCAT is its “multi-chain comparison mode” (see Modularized algorithms and algorithm chain Section), in which multiple algorithms chains are compared for the given problem. b) Annotation and classification of image sets or ROIs, using the chosen model. BIOCAT can work with both image classification or multi-label annotation: If each image in a set corresponds to one label, then it resembles normal image classification; If each image could correspond to more than one labels, then it is a multi-label annotation task.

In order to facilitate supervised learning, the training images (or regions) are associated with corresponding labels. BIOCAT allows three possible ways of such association and the user can pick the most suitable way for him/her: a) target file input mode: a text file that assigns a label or multiple labels to each image; b) directory tree input mode: a directory structure with each subdirectory being an image category; or c) ROI mode: ROI/landmark files where the name of the ImageJ ROI zip file or Vaa3D landmark file indicates the image category.

#### Extensibility

BIOCAT has a modular design, and thus is extensible and developer-friendly. BIOCAT algorithm specification is provided for extending BIOCAT with more algorithms: An algorithm developer can add a new algorithm to BIOCAT as long as it conforms to the API (Application Programming Interface) specifications for a feature extractor, a feature selector, or a classifier. An XML file can be used by the module developer to specify parameters of the algorithm that will show up on GUI. (See Developer’s Guide on BIOCAT website for tutorial).

#### Inter-operability

BIOCAT runs as a standalone application that focuses on classifying/annotating a set of images (or ROIs in them) using pattern recognition algorithms. It can inter-operate with other tools with different focus. The ROI manager in ImageJ and landmark manager in Vaa3D can be used to save ROIs and import them into BIOCAT for training. Classification/annotation output files can be edited to be Vaa3D landmark files for further visualization. BIOCAT also generates reports in PDF files for algorithm comparison results and annotation results.

#### Portability

BIOCAT is developed in Java. Thus this software is portable to various operating systems such as Windows, Mac and Linux as long as Java Virtual Machine is available.

To summarize, the uniqueness of the BIOCAT tool, other than the list of built-in pattern recognition algorithms suitable for image classification tasks (to be explained in next section), is that the algorithms are modularized so that they can be “chained” in a customizable way to form adaptive solution for various problems, and the plugin-based extensibility gives the tool an open and flexible architecture to include future algorithms.

### Modularized algorithms and algorithm chain

The BIOCAT’s algorithms are modularized to facilitate the adaptive comparison of what we call “algorithm chains”, which is a sequence of several pattern recognition algorithms that include some feature extractors, one or more optional feature selector(s), and a classifier.

For feature extraction, we consider 2D and 3D textural, morphological and structural features. Texture features are an important category of effective image features such as wavelet features. Morphological features describe the shape and structure of 2D and 3D objects. A pixel in a 2D digital image consists of 4 or 8 neighbor pixels depending on whether the diagonal neighbors are considered. Some 3D features can be extended from 2D by extending an 8-connected neighborhood to a 3D 26-connected neighborhood. Examples of morphological features include Hu moments, which are statistical summaries of intensity in the 2D or 3D object neighborhood, and measures in relation to the center of mass of the images [28] or Zernike moments which are orthogonal invariant moments using a set of complex polynomials [29]. Other algorithms include Hessian features which are suitable for detecting tube-like neuronal structures, Gaussian derivatives and Laplacian features [30], as well as object statistics [21]. Currently 14 different feature extractors are available in BIOCAT with the following examples, each providing one to multiple measures extracted from an image:

•8 2D Hu Moments, invariant to translation, scale and rotation.

•8 3D Hu Moments: 3D extension of 2D Hu Moments;

•Zernike moments: Default to the first 20 Zernike moments.

•7 statistics for the objects in an image: number of objects; their average size and variance, and their spatial location in relation to center of mass of the image, both average and variance; ratio of the size of the largest object to the smallest; ratio of the distances to center of mass between the furthest and the closest.

•Object territory: overall territory the foreground object.

•Image’s Hessian and Laplacian features.

•2D and 3D wavelet features. The 3D anisotropic wavelet texture feature will be detailed in 3D Anisotropic Wavelet Texture Feature Section.

BIOCAT has feature selectors such as Fisher’s criterion that select a subset of features. BIOCAT also provides several classifiers including support vector classifiers [31], nearest neighbor, Naïve Bayes, decision tree, and random forest.

As explained in Implementation Section, BIOCAT supports an extensible design, so the above algorithms are of a growing list. Currently, BIOCAT has about 20 algorithms for 2D/3D feature extractors, selectors and classifiers. The list includes the 3D anisotropic wavelet feature extractor and re-implementations of known algorithms (a majority of the feature extractors are re-implementations). The (re)implementations are memory- and efficiency-aware for working with high content multidimensional bioimages. These feature extractors in BIOCAT reuse the memory space for each image. In addition, due to the extensible design, libraries may be wrapped and incorporated as plugins whenever such algorithms become available. For example, Hessian features is a plugin wrapped around FeatureJ implementation [30].

Note that the “algorithm chain” in our paper refers to a sequential flow of feature extraction, selection and classification, which is different from the narrower sense of supervised (or unsupervised) learning of pattern recognition. When multiple feature extractors are used, these features are linearly aggregated. The combined feature’s dimensionality is the sum of all individual feature vectors. Such combination may lose some property (e.g. invariance) of certain features if they are not combined with features with similar property, yet it provides the flexibility for extending to many feature extractors that are often useful for biological image classification. When multiple selectors are used, it is a cascading design: the feature vector goes through the first selector. The resulting feature vector is then filtered by the second selector and so on.

With all the algorithm modules as building blocks, BIOCAT can conveniently build algorithm chains to compare multiple alternative models. One example algorithm chain can be an anisotropic 3D wavelet feature extractor along with a 3D Hu Moments feature extractor, followed by a Fisher feature selection and ultimately a support vector classifier. The chains with the same types of algorithms but different parameters are considered to be different chains, so that dissimilar parameters (e.g. multiple wavelets) can be compared to find the suitable ones for the model. Figure 1b shows BIOCAT’s screenshot of building and comparing the algorithm chains.

In addition, functionality is included in BIOCAT for ROI annotation (Here the term “annotation” refers to the computational task of automatically labeling a new image which is formulated as a supervised pattern recognition task):

1. Sliding window-based ROI annotation. With a model learned and selected, BIOCAT can conduct automatic ROI annotation on a new image using a window sliding algorithm. A sliding interval parameter is defined which represents how many pixels apart will the trained model be applied to a pixel/voxel. Because it represents the frequency of decision making, a small interval leads to a higher annotation resolution with longer running time. When the interval is set to 1, then every pixel in the image is annotated: a ROI is extracted surrounding each pixel/voxel, and a classification is performed to assign a category to the pixel/voxel. The parameter can be adjusted via GUI.

2. In the case of 3D image annotation, it is possible that a large portion of the 3D image is black. To improve annotation efficiency, local maxima can be calculated using morphological dilation. The decision can thus be made only on the set of local maximum voxels. Such scenarios are useful for 3D object quantification (objects are labeled based on the pixel classification of BIOCAT), such as synapse or cell counting using 3D confocal images, where objects of interest are the bright regions in largely dark images.

### 3D anisotropic wavelet texture feature

One important category of effective image features is texture features, which is a family of features that measure the texture of images such as wavelet features. In particular, wavelet features are obtained using discrete wavelet transform (DTW) with wavelet functions [32]. Two dimensional wavelet features had performed well in our previous work with gene-expression annotation of fruit fly [16]. However, full extension of these features from 2D to 3D can lead to a big increase in the number of features, and consequently the storage and computational need. For example, for wavelet transform, a full extension to 3D wavelet features will lead to a number of features that is cubic to the side length of the object (instead of being quadratic in 2D). We thus designed a multi-scale anisotropic 3D wavelet feature. Such features can be particularly suitable for 3D confocal microscopic images since it adapts to the anisotropic nature of confocal imaging where z resolution is typically less than x-y resolution.

The feature for a voxel of interest at (x,y,z) is extracted from its surrounding 3D volume:

$$f_{k,n}\left(x,y,z\right)=\overset{z+\mathrm{rz}}{\underset{z_{i}=z-\mathrm{rz}}{\sum }}w\left(z_{i}\right)\overset{y+\mathrm{ry}}{\underset{y_{i}=y-\mathrm{ry}}{\sum }}\overset{x+\mathrm{rx}}{\underset{x_{i}=x-\mathrm{rx}}{\sum }}\psi_{k,n}\left(x_{i},y_{i}\right)I\left(x_{i},y_{i,}z_{i}\right)$$

where *I* is the intensity, the size of the extracted volume is (2*rx+1)*(2*ry+1)*(2*rz+1), ψ is the discrete Haar wavelet basis function, *k, n* are the dilation and translation factors of wavelet with k=0,1 (for two-level wavelets), n=0....(2*rx*+1)*(2*ry*+1), and (2*rz+1) is the number of slices included in calculation. *w*(.) is a Gaussian function with middle z slices weighted heavier than other slices.

**Figure 2:**
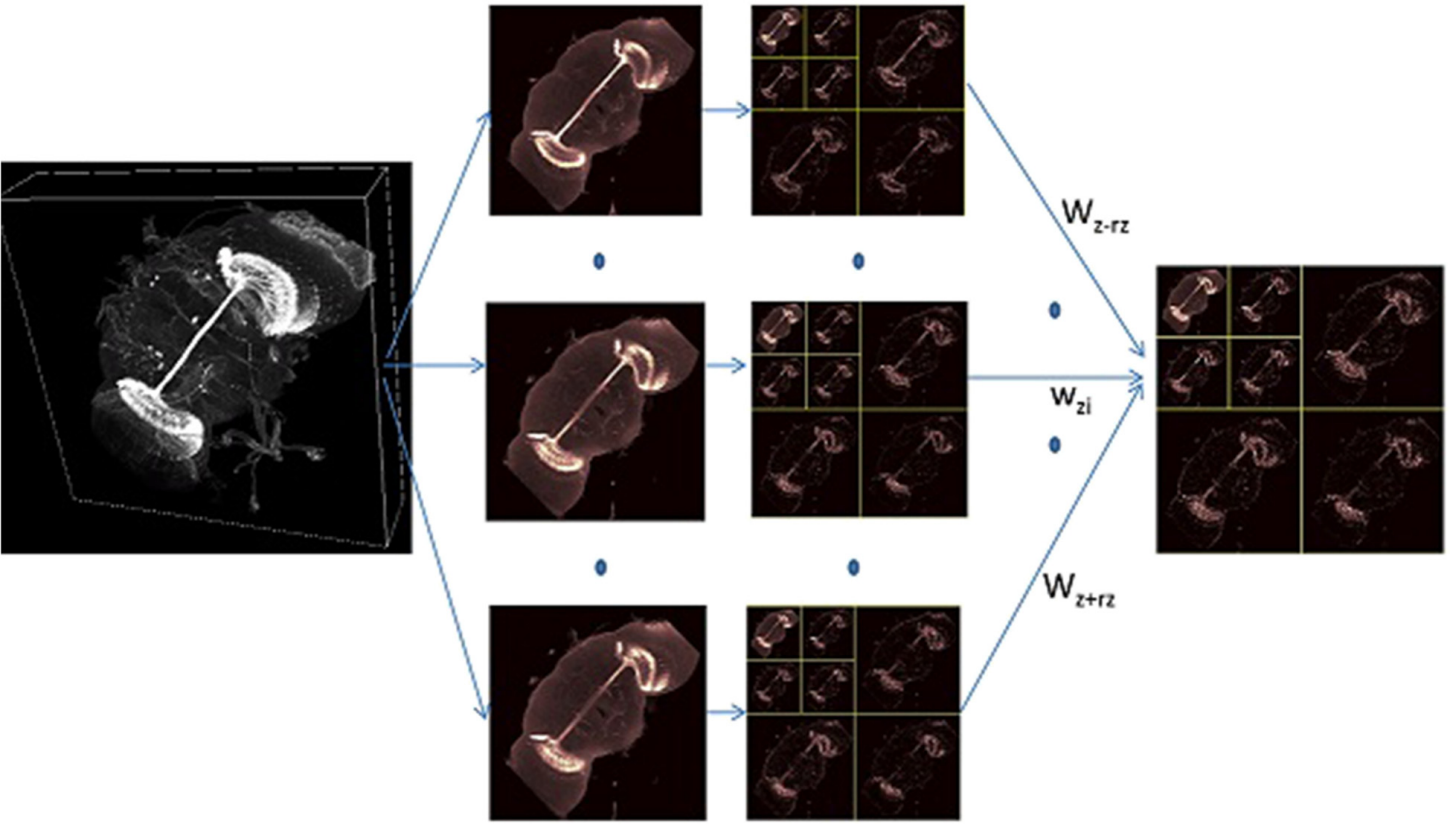
An example of extracted isotropic wavelet features from a 3D neuronal image.

The multi-scale component of the 3D anisotropic wavelet is represented by the dilation factor k of the wavelet transform. The number of the scales is also called wavelet level. At each level, a Haar-based discrete wavelet transform is done on the approximation image from the previous finer resolution. Figure 2 shows an example of level-2 3D isotropic wavelet, where 2*rz+1 slices are involved in calculating the feature for a 3D image of a fluorescently labeled fruitfly brain.

Note that other tools also use wavelet for image analysis. For example, Icy [11]’s spot detector plugin [33] uses wavelet adaptive threshold for object detection, which is an unsupervised scheme different from the method presented in BIOCAT.

## Experiments and results

BIOCAT is able to associate a biological image to one of many categories of interest, and perform 2D/3D ROI annotation based on ROI classification, as well as multi-label image annotation. This section shows experiments on using BIOCAT for these tasks and demonstrates the effectiveness of the algorithm modules as well as the automatic algorithm selection conducted by BIOCAT. These case studies are also selected due to their diversity. In particular, 3D ROI Classification and Quantification section and 2D ROI Classification and Quantification section describe how BIOCAT can assist in some popular quantification problems encountered in cell biology and neuroscience.

### 2D/3D image set classification and model selection

We tested BIOCAT with thirteen biological image sets for classification. Additional file 1: Table S1 shows the properties of the image sets, which shows that the image sets are from different sources with various imaging modalities and characteristics. Among the image sets, K150 2D and K150 3D are fluorescent-labeled confocal microscopic images of fruitfly brain for expressed neuronal bundles of different genetic lines. Others are image sets corresponding to different types of sub-cellular locations (e.g. CHO) or different cellular types (e.g. Binucleate) [21]. When classifying an image, if the image contains multiple channels (as is often the case with fluorescent stained microscopic images), BIOCAT can work on a selected channel. For k150 2D and 3D image sets which are three-channel RGB images, the classification is done on the green channel which corresponds to the GFP expression. For data sets do not separate between training set and testing set (all except k150), we report the recognition rates using five-fold cross-validation. We repeat the cross-validation for five times (by randomly shuffling data) for a total of 25 runs. The mean and variance of recognition rates over the runs are computed and reported in Table 2.

**Table 2:** Image set classification results of BIOCAT

**Image set**	**Literature accuracy (%)**	**BIOCAT accuracy (%)**	**BIOCAT variance**	**BIOCAT number of features per image**
K1502D	-	**100**	-	40
K1503D	-	**100**	0.0	500
CHO	93	**93.1**	0.12	7
Binucleate	100	**100**	0.0	40
LiverGenderCR	99	**99.0**	0.002	40
LiverGenderAL	69	**91.7**	0.65	100
MuscleAge	53	**89.6**	0.10	100
Pollen	**97**	87.5	1.15	40
Hela	**84**	68.3	0.15	40
Termbulb	49	**51.1**	0.31	21
Lymphoma	**85**	70.9	0.07	22
LiverAging	51	**73.8**	0.81	14
RNAi	**82**	55.0	0.08	40

The bold numbers indicate the higher accuracy.

Table 2 also gives the comparison of performances between BIOCAT and the benchmarking results in literature. Literature results are from the Wndchrm tool which achieves the best accuracy on the sets so far. Wndchrm has a fixed option of extracting about 1000 or 2600 features regardless of the problem. For comparison, we also report the number of features used by BIOCAT in Table 2.

Results in Table 2 show that BIOCAT can effectively classify various biological image sets. For nine out of the thirteen data sets, BIOCAT achieves the state-of-the-art or better accuracy. For Binuleate and LiverGenderCR, only 40 features per image were used to achieve about 100% accuracy. For MuscleAge, LiverAging and LiverGenerAL sets, BIOCAT is able to greatly improve the current best results. For the CHO set, by using object statistics alone, which is a set of 7 features, BIOCAT achieves comparable results with Wndcharm and 6.3% more than the original literature (Random Forest is used as the classifier). Comparatively, Wndchrm needs a much larger number of features to achieve the reported results. Wndchrm’s need of calculating thousands of complex features can lead to a much higher computational complexity which would make it slow on larger images (Wndchrm does not have a classification functionality for 3D images which would be more computational demanding.) BIOCAT’s adaptive design, on the other hand, allows the best suitable algorithms to be selected for a given biological image classification problem, which can sometimes be simple, as in the case of the CHO problem.

We also see that BIOCAT does not outperform literature on four sets of Pollen, Hela, Lymphoma and RNAi. It may be due to that reported results are based on current built-in algorithms in BIOCAT and Wndchrm’s longer list of descriptors including Halalick textures could have helped on these specific sets. Further effort on parameter fine tuning may also help improving the results. In this paper, we have been focusing on demonstrating the tool’s capability of empirical model comparison. Given the extensible design of BIOCAT, we expect to continue improving the accuracy as new algorithm modules suitable for the problems being added in.

On the other hand, it has also been pointed out that some low level descriptors could cause bias on classifying some biological images sets [34]. For example, CHO and Binucleate datasets can be biased in the sense that background artifacts could contribute in classification [34], so the accuracies may be optimistic. In general, it is worth noting that features and results should be validated in terms of biological relevance and best practice on this regard will be further studied.

In Additional file 2: Table S2, we demonstrate the 3D anisotropic wavelet feature for the K1503D image set, and how BIOCAT was used to do chain comparison. The anisotropic wavelet feature has rz set to 1 (3 neighboring z-slices are used). Wavelet level is set to 2. The nearest neighbor is a 3NN. Support Vector Machine classifier has a linear kernel and the regularization parameter set to 1. Fisher selector selects the best 500 features. As we see from Additional file 2: Table S2, the three algorithm chains containing 3D anisotropic wavelet (chains 1–3) deliver very satisfactory results. Both chain 1 and chain 2 achieve 100% accuracy with SVM as the classifier, regardless of whether Fisher’s feature selection is performed. Chain 3 has 90% accuracy with Random Forest as the classifier, while the alternative feature Hu Moments has a mediocre performance compared with Anisotropic wavelet even when the same type of classifier is used (Chain 4). This shows the potential effectiveness of the anisotropic wavelet feature as a 3D feature extractor. The bar chart that compares and reports the results of the five chains is directly from BIOCAT’s GUI, as part of its model selection functionality.

**Figure 3:**
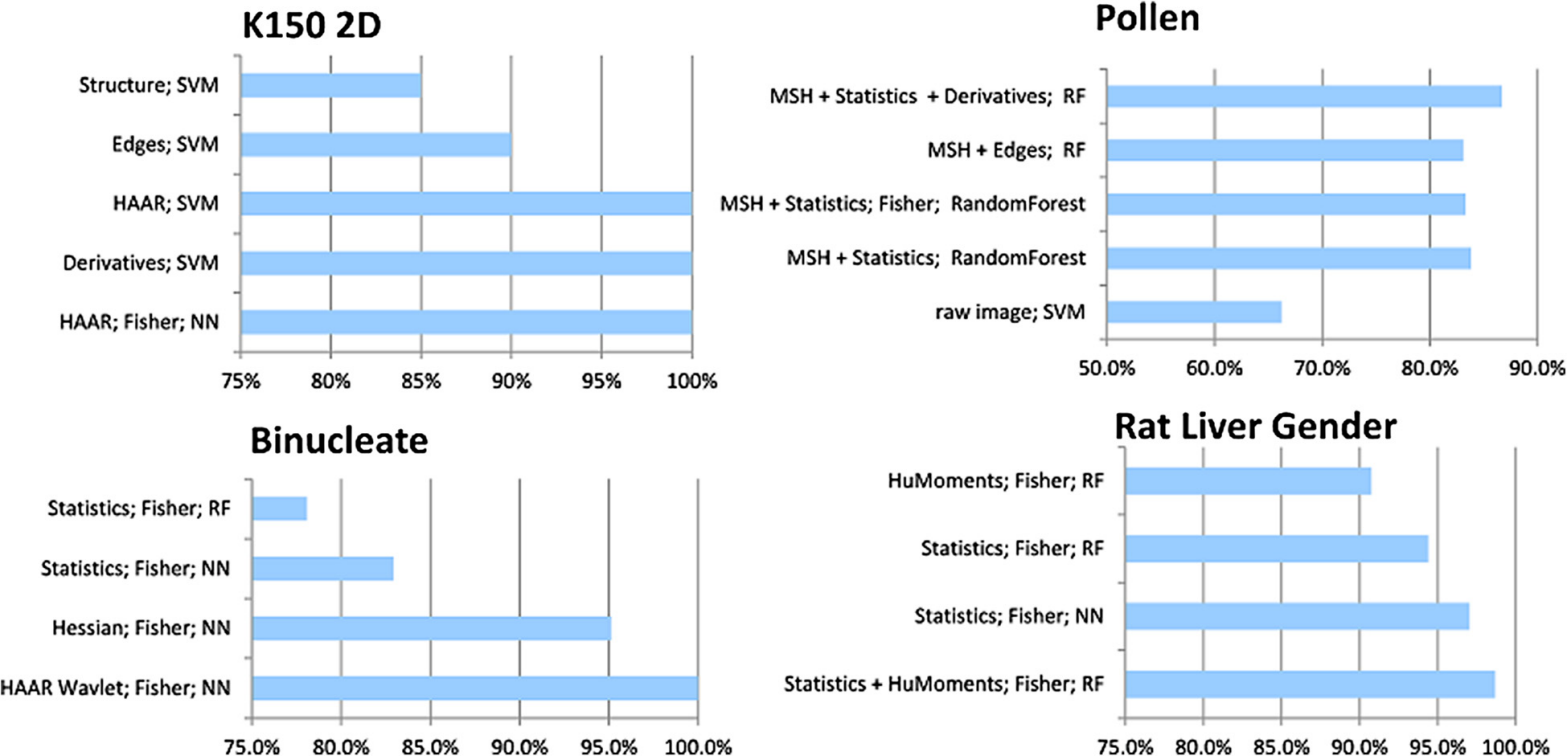
**BIOCAT performs algorithm chain comparison adaptively for different data sets.** Acronym: RF: Random Forest Classifier; NN: Nearest Neighbor Classifier. MSH: Moments (Hu) + Structure Features + HAAR Wavelets. Default parameters are used for all the algorithms unless otherwise specified.

Figure 3 further exemplifies how BIOCAT selects an effective chain on some image sets in Additional file 1: Table S1. For each dataset, BIOCAT dynamically looks for a suitable combination of feature extractors, selectors and classifier, chooses the most effective algorithm chain for the given image set among candidate chains. For example, for the Binucleate set, the algorithm chain of “HAAR extractor; Fisher selector; 3-Nearest Neighbor classifier” out-wins other chains and gain 100% accuracy.

### 3D ROI classification and quantification

3D ROI quantification such as cell counting in high content images is a common problem in biology. Traditionally, it has been done using approaches such as connected component analysis or template matching without training a classifier. The general consensus on supervised learning using of a trained model has been that it can produce robust quantification results [15-19,21,35] and is suitable for large-scale analysis due to minimal user intervention [36]. Recent visualization tools such as Vaa3D has also made tagging of the 3D images more convenient (thus creating the training set is easier). BIOCAT can further facilitate pattern recognition based approach for 3D ROI classification and quantification.

**Figure 4:**
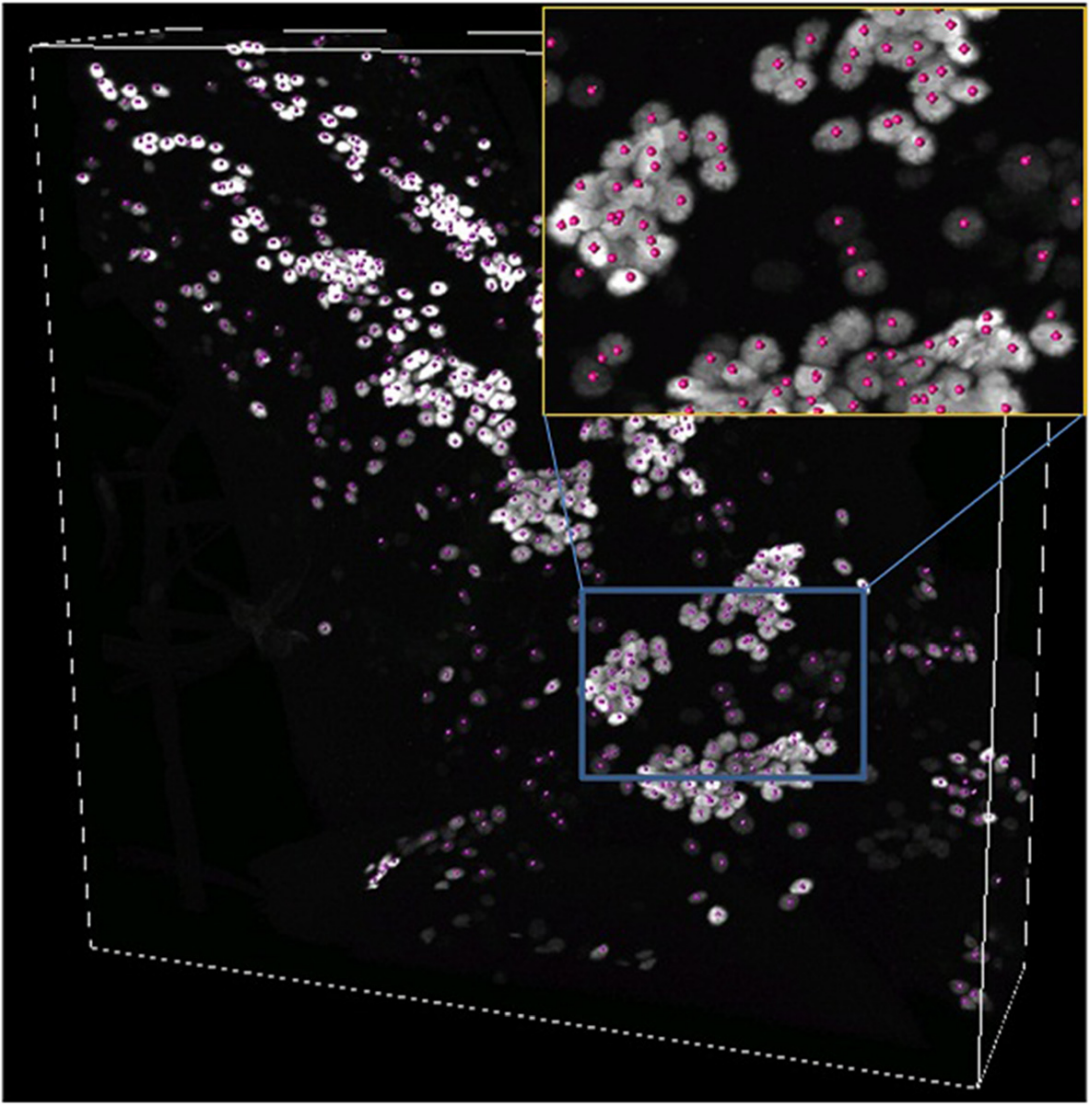
**A 3D confocal image of fruitfly larvae neurons (the channel of cell nuclei).** Red dots are the marked centers of nuclei. Dimension is 512*512*148.

Figure 4 shows an example of neurons in *Drosophila m.* (fruit fly) larvae nervous system, where gray clusters are the nuclei. Such images may contain thousands of cells per image. Often the cells form crowded clusters and the boundary among the objects may be blurry (see the zoomed area in Figure 4), which make such object counting in 3D biological images a challenging task.

We formulate the object counting as a pattern recognition problem based on voxel classification: For each voxel, we develop a model using BIOCAT to detect if it is potentially a center of a nuclei. A training set for ROIs on a training image with associated labels (e.g. positive and negative ROIs) needs to be generated. The process for 3D ROI classification then starts with loading the image and labeled ROIs into BIOCAT for running the model selection. Once the model is picked, new images are loaded into BIOCAT, ROIs are classified. Classification can be limited to images’ local maxima if needed.

Additional file 3: Table S3 shows the example algorithm chains compared during the model selection process of BIOCAT for the case study. We labeled 560 positive voxels and 430 negative voxels in the image. The 3D ROI volume is 7*7*5. The rz in 3D anisotropic wavelet is set to 2. SVM classifier uses linear kernel with regularization parameter set to 1. Five-fold cross validation is used to report the results. Chains are built and compared by BIOCAT to select effective models for the classification of 3D ROI around a fruitfly nuclei center. We experiment the cases when only 3D Anisotropic wavelet features are used; only 3D Hu Moments features are used; and the combination of 3D Anisotropic wavelet and Hu Moments features are used.

Additional file 3: Table S3 shows that 3D anisotropic wavelet feature alone can effectively yield 98.6% recognition rate. On the other hand, if only 3D Moments are used as the features extracted from the 3D ROI surrounding the voxel, recognition rate is only 96%. The combination of both features can further improve the recognition rate to 99.2%. It again shows that the 3D Anisotropic wavelet may be an effective 3D feature extractor for biological image classification. Additional file 3: Table S3 also shows that the use of random-forest classifier or support vector machine yields about the same result. All the comparisons among algorithm chains listed in Additional file 3: Table S3 are done by BIOCAT and the bar chart is the output of the tool.

The predicted center candidates by BIOCAT can be exported. They may be used for further quantification purposes. For example, the cell counting application described in [17] does neuron counting by performing mean shifting on the detected centers to move them to the closest center of mass. The nearby centers are merged before yielding the cell count. The advantage of such cell counting based on ROI classification is that when the cells are largely clustered and the boundary are blurry (which can be common in biological objects such as cells), the approach can give a count without the need of segmentation, especially when the shape of the object is oval.

### 2D ROI classification and quantification

To demonstrate BIOCAT’s 2D ROI classification, we describe a case study of axon detection for neuron morphology profiling in this experiment, in which we employ BIOCAT to detect axon pixels from the neuronal image to assist quantification of neuronal dendritic territory.

**Figure 5:**
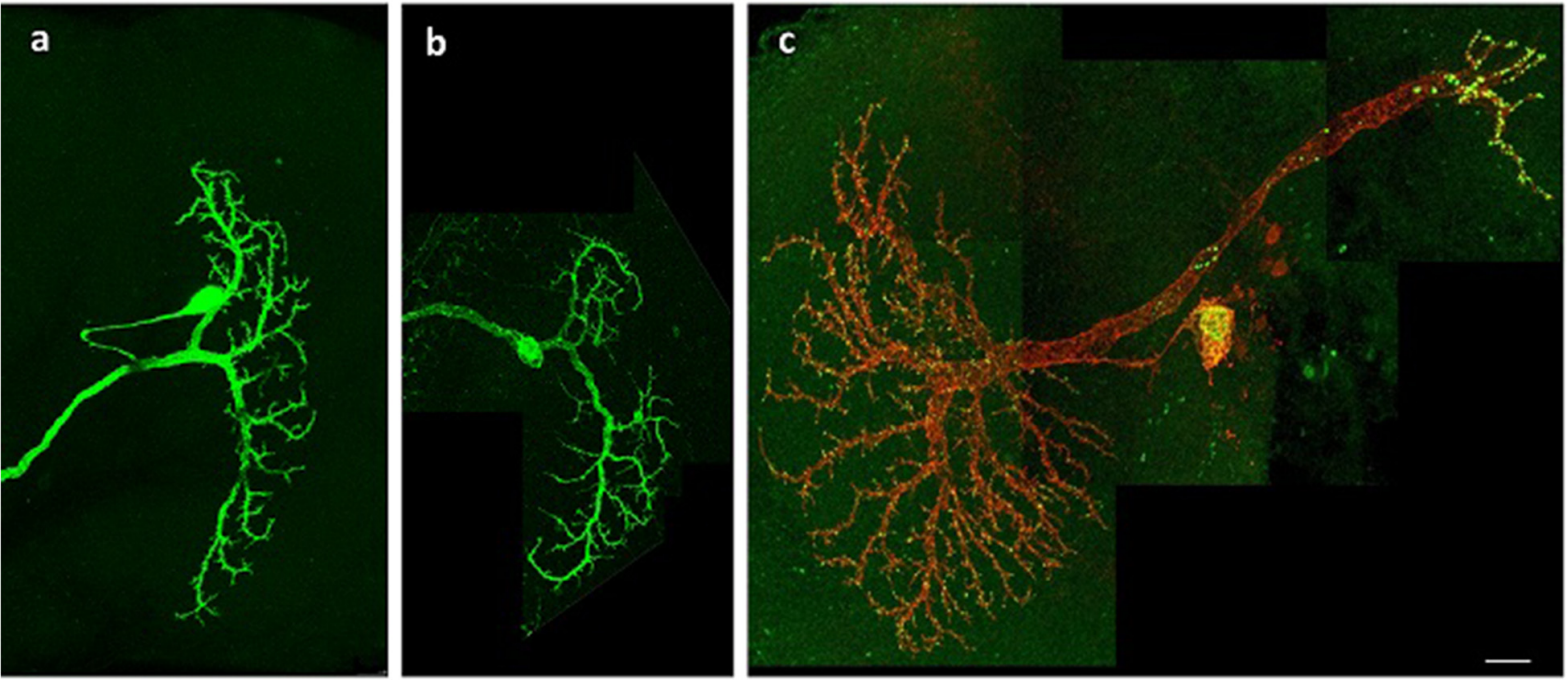
**Example images of case applications. a)** A wild type LPTC Vertical System 1 (VS1) neuron in a fruitfly adult brain; **b)** A mutant of VS1 neuron. Neuron morphology of **a)** and **b)** are labeled using green fluorescent protein; **c)** A LPTC Horizontal System (HS) neuron whose morphology is labeled with a membrane-tethered red fluorescent protein. Images shown in **a**, **b**, and **c)** are maximal projections of 3D image obtained by laser scanning confocal microscopy. Scale bar: 10 microns.

Intricate morphology is a striking feature of neurons and plays an important role in functional analysis and quantification of neuronal systems [37]. Among the neuronal morphometrics, the territory occupied by the dendritic tree is an important measure of the neuron morphology that not only indicates defective shapes of potential (dys)function of neurons, but also serves as an important parameterization factor for further quantification analysis in mutant screening. We consider GFP-labeled lobula plate tangential cells (LPTCs) in the brain of the adult *Drosophila m.* (fruit fly) (Figure 5). To extract the dendritic tree, we first automatically detect and remove non-dendritic subcellular components (soma and axon) from the image. Upon removal of the axon and soma, the territory of the dendritic tree can be easily estimated. In this paper, we focus on explaining the use of BIOCAT for axon detection: Similar as 3D ROI annotation, the flow starts with tagging the ROIs for training, then BIOCAT employs a trained model to detect axon candidate pixels from the neuronal image.

**Figure 6:**
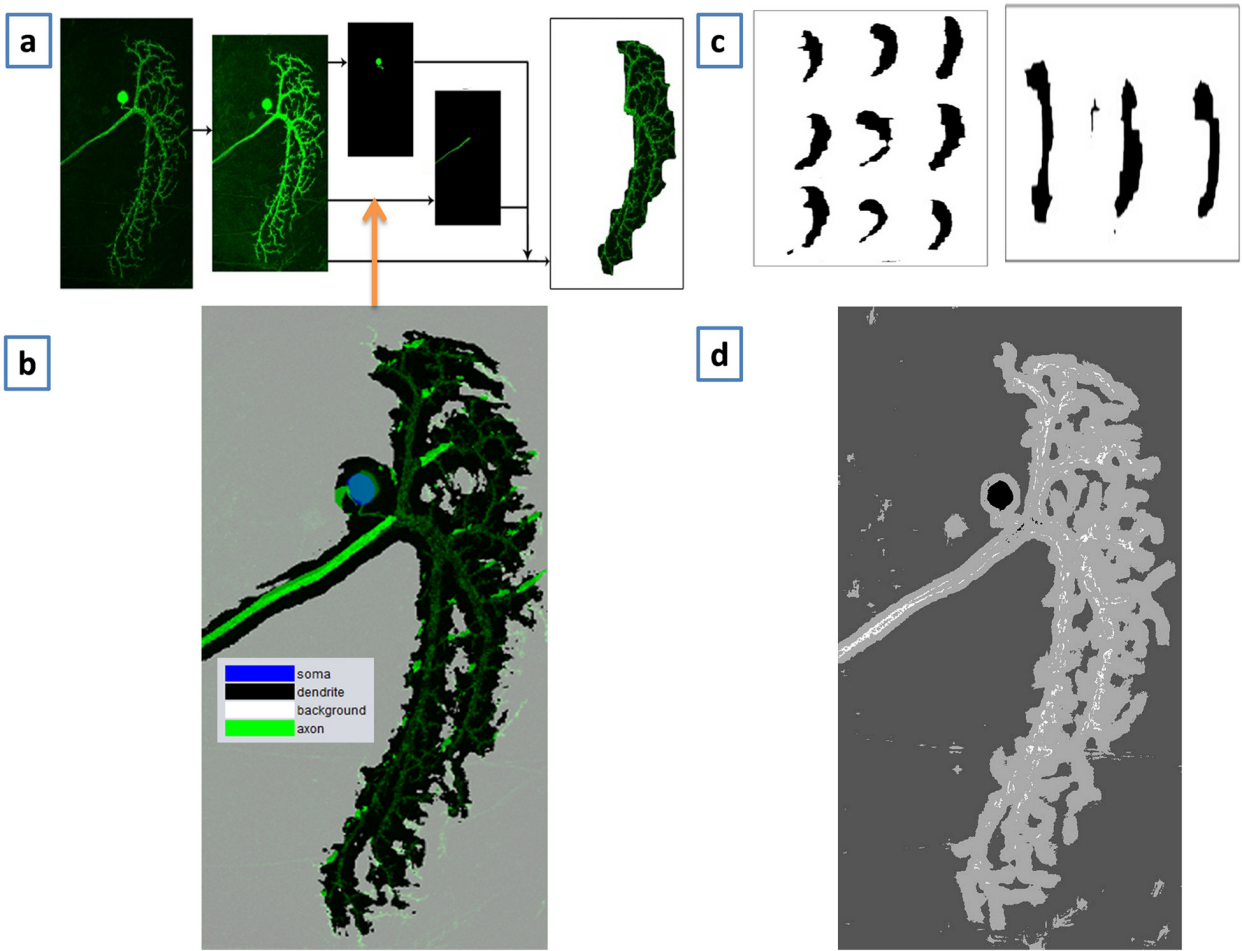
**Territory quantification for wild type and mutant type neurons assisted by BIOCAT. a)** The territory extraction where axon removal is assisted by BIOCAT. **b)** BIOCAT’s output of ROI annotation (overlay on the original image.) **c)** Examples of extracted territories for wild type and mutant neurons; **d)** ROI annotation from a Fiji plugin “trainable segmentation” [38].

A training set consisting of 4 categories of image regions are randomly extracted from two neuron images – one wild type and one mutant type. The training set includes 10 axons, 50 branches, 7 soma and 9 background image regions. We make use of BIOCAT, which performs comparison of various combinations of extractors, selectors and classifiers as explained in previous sections. The winner algorithm chain consists of 2D HAAR discrete wavelet as features, Fisher’s criterion that selects top 40 features, and the Support Vector Machine as the classifier. Once the model was built, it was applied to other LPTC images to automatically annotate image regions and export the regions identified as axon candidates. Figure 6b visualizes how BIOCAT can be used to annotate axon, dendrites, soma and background. It is an overlay of the annotation results on the original image: The original neuron was light green; The axon and soma pixels are annotated using bright green and blue, respectively. A sliding window algorithm with interval parameter being 1 pixel is used in annotation. The axon candidates detected by BIOCAT will then be post-processed by finding the area using length and orientation considering that the axon is a long tubular structure. Once the axon (and soma) is removed, a rolling-ball algorithm is done to estimate the territory of the dendritic tree. Figure 6c shows the results of extracted dendritic territories from wild type and mutant neurons.

In this experiment, BIOCAT plays a similar role as segmentation, except that most segmentation tools deal with two classes (foreground and background), while the ROI classification gives multiple classes to annotate a pixel/voxel. We used a Fiji plug-in, “trainable segmentation” [38], to compare the results of the multi-class annotation for the given image. The plugin uses a trainable model (a fixed set of features and random forest classifier) to classify each pixel of the image. Figure 6d shows its output image, where the 4 gray levels, from deep to light, represent soma, background, dendrite and soma, respectively. We can see that although the plugin can also detect soma, it is less effective in separating axon from dendrite (many axon pixels are classified as dendrites). BIOCAT can extract the entire axon (the bright green region in Figure 6b. We can also see that BIOCAT classifies the background pixels accurately as indicated by a cleanly labeled background region (because Figure 6b is an overlay of output and original image, the annotated background shows as gray).

## Discussions

### Adaptive algorithm chain selection

BIOCAT provides a flexible and adaptive platform that accommodates the ever-growing image samples and emerging pattern recognition and machine learning algorithms. Table 2, Additional file 2: Table S2 and Figure 3 show that different algorithms and their combinations indeed have different suitability for various problems. For example, in Figure 3, we see that while the random forest classifier does not perform ideally for the Binucleate set, it is effective for the Rat LiverGenderCR set (both with default parameter setting). Similar variations of efficacy can be found in feature sets.

The advantage of such an adaptive design is to find a good set of features for a given problem, without paying the cost of extracting a huge set of features or using a unsuitable classifier. Current related tools exploit two major approaches: 1) Use a fixed model including a feature set of medium size; 2) Start with a huge feature set, gaining accuracy at the cost of computational complexity. The former typically works better on some problems but not others. The latter can be an overkill on some cases and not suitable for large scale images due to computational complexity. For example, Wndchrm extracts thousands of features for any problem, among them Zernike features has a factorial complexity due to its radial polynomial coefficients. Some features are extracted using transforms based on the result of other transforms which are also very computationally demanding.

BIOCAT takes a different approach. It provides the platform of adaptively building different models for different problems, which may lead to fewer feature descriptors that are yet effective for classification. Note that such model selection is different from the computational selection of feature columns using statistical or other criteria as what Fisher’s (or similar) feature selector would do. It is because feature selectors just select indexed columns for a large matrix which may belong to different extractors. As a result, the annotation/testing stage would still need to start with *all* the feature extractors that are involved, which can negatively impact the performance for large images/sets. On the other hand, such features selectors can of course be part of an algorithm chain for BIOCAT, to further reduce the bandwidth for the classifier. Such approach also brings a better interpretability, which may potentially lead to a reversed way to understand some mechanism of differentiation among different biological structures or developmental stages.

BIOCAT also allows the selection of a suitable classifier as part of the algorithm chain, which few other tool for biological image classification offers (see Table 1). Instead of having computer scientists comparing (and often re-implementing) various classifiers, it provides biologists a GUI-based and objective approach to find a good pattern recognition model. BIOCAT GUI also allows to build the algorithm chains incrementally, to save/load chains and for generating reports after the chains are compared.

### Speed and computational complexity

BIOCAT is able to process the datasets in Additional file 1: Table S1 within a reasonable period of time. The speed ranges from several seconds to several minutes with the sets on a typical PC (Intel Core i5 2.67 GHz 8 GRAM).

At the framework level, BIOCAT is designed to be memory-aware. Specifically, BIOCAT’s built-in feature extractors run on the image set in sequential fashion to reduce the memory footprint. In the case of 2D image sets, one image is loaded at a time. In the case of 3D images, one slice of an image is loaded at a time. After features are calculated, the same memory space is reused by the next image/slice. Since only the most current stack slice of the most current image is cached in memory, BIOCAT has a low requirement of computer memory at feature extraction stage. After feature extraction, subsequent feature selection and classification’s memory requirements are affected by image set size and feature set size (but not the original image size). We need to point out that individual algorithms have different levels of memory requirements and some third-party algorithms (e.g. Weka classifiers) may require bigger memory to process large sets. The speed of model comparison is linear with the number of models to be compared.

### Multi-label annotation

In Experiments and Results Section, we demonstrated annotating an image by classifying ROIs surrounding a pixel/voxel. In addition, an entire image can also be automatically annotated, by formulating the task into a pattern recognition problem. Such annotation often involves multi-labels per image since an image may have multiple tags to be assigned. Annotation of biological images has traditionally been manual work, done by domain experts in a labor-intensive way. Recently, automated annotation has been attempted [16,25,26,39].

The difference between multi-label annotation and the usual image classification is that typically an image classification task is single-objective (e.g. an image or a ROI corresponds to one and only one category in classification), whereas an automatic annotation task can be multi-objective. For example, fruit fly embryonic gene expression involves the annotation of multiple developed body parts in each gene expression image [16]. For such cases, BIOCAT chooses to formulate the annotation task as multiple binary problems, with each problem focusing on whether or not a label is present. Such labeling can be described using a text file (“target file” as mentioned in 3D Anisotropic Wavelet Texture Feature Section), which associates a training image with multiple labels. Model selection can be conducted for each sub-problem individually. During annotation, the collection of learned models, each for a label of interest, will be applied to the testing image. BIOCAT’s reporting tool outputs a consolidated annotation report, summarizing each testing image’s labels, ranging from zero to many. BIOCAT’s website shows an example and screenshot of such multi-label annotation task.

### Connection with other tools and limitations

BIOCAT is designed with different purpose from tools such as ImageJ (for image processing) or CellProfiler (mostly for 2D cell segmentation/measurement). It does not reinvent the wheel. Instead, it focuses on the area where current tools are lacking, and meets the need of adaptable and extendable pattern recognition algorithms for image and ROI classification. On the other hand, they can certainly work together to complement each other. For example, ImageJ can be used to label ROIs in order to generate training sets for BIOCAT, or for preprocessing before the images are being classified. Other visualization tools such as Vaa3D can also be used for 3D ROI labeling and visualization, to complement BIOCAT.

ROI annotation is not the same as segmentation: For example, in the 3D ROI classification example, the cell counting may be done without knowing the exact boundaries of objects. Segmentation in a traditional sense is typically the issue of separating foreground from background and often unsupervised, while BIOCAT can work with multiple classes for the purpose of labeling/annotation. Segmented images by other tools such as ITK (Insight Segmentation and Registration Toolkit) can be used as input for classification by BIOCAT, while BIOCAT’s detection results may also be post-processed to deliver results similar as segmentation when every pixel/voxel is annotated.

BIOCAT is also different from general sense pattern recognition tools in that the built-in algorithms pay more attention to efficiency and memory since an image typically contains large number of pixels which is the raw dimension of data. It also has other functionalities specifically related to biological images. For example, if the image contains multiple channels, as is often the case with florescent stained microscopic images, BIOCAT can work on a selected channel. It can also limit the annotation of images to local maxima, useful for 3D ROI classification in fluorescent stained microscopic images. Its extensible design, on the other hand, can facilitate the inclusion of state-of-the-art algorithms in the fields of pattern recognition and machine learning, as they appear.

BIOCAT presents several limitations (or things it is not designed to do): There are no image processing algorithms in BIOCAT such as denoising or enhancement and the tool’s output often needs further post-processing to get results for the specific quantification. So BIOCAT is not intended as a replacement for either image processing or segmentation tools. Instead, it is for choosing a suitable supervised pattern recognition model, which can then be used as a discriminative model in classifying 2D/3D image sets or ROIs. The tool currently does not do exhaustive search of all combinations of algorithms for the consideration of computational feasibility. As the result, the selected chain is the best among all compared chains, but not necessarily optimal. It is also noted that some machine learning algorithms can be slow. When the user works with a large image set with high dimensionality, a GUI-based tool on a PC is not always the best choice. HPC version of BIOCAT for distributed model selection is currently under development.

## Conclusions

BIOCAT generalizes pattern recognition based image classification to three dimensional images and ROIs and provides a comparison mechanism among algorithms. It provides good flexibility and adaptability compared to most related tools, which we expect to facilitate the use of pattern recognition algorithms in a range of biological problems. For future directions, more algorithm modules are being developed and a version of BIOCAT for cluster computing is also under development for very large biological image sets.

## Availability and requirements

•**Project name:** BIOCAT

•**Project home page:**http://faculty.cs.niu.edu/~zhou/tool/biocat/

•**Operating system(s):** Platform independent

•**Programming language:** Java

•**Other requirements:** Java 1.6.1 or higher

•**License:** FreeBSD

### Availability of supporting data

The software, along with supplementary materials can be found at http://faculty.cs.niu.edu/~zhou/tool/biocat/. The example images sets are available for download either from BIOCAT website (e.g. k150) or from the original provider’s site.

## Competing interests

The authors declare that they have no competing interests.

## Authors’ contributions

JZ designed the project, implemented BIOCAT, performed most experiments and wrote the paper, SL implemented BIOCAT and performed the experiments on dendritic territory extraction, GS and BY designed the dendritic territory experiment and performed imaging, contributed to the algorithm design and revised the paper, HP conceived the project and contributed to the initial design of BIOCAT and revised the paper. All authors read and approved the final manuscript.

## Supplementary Material

Additional file 1: Table S1 Biological image sets. Click here for file

Additional file 2: Table S2 Algorithm chain comparison algorithm chains for K150 3D. Click here for file

Additional file 3: Table S3 Algorithm chain comparison for classification of 3D ROI around a fruit fly nuclei center). Click here for file

## References

[B1] Pawley JB Handbook of biological confocal microscopy, 3rd ed 2006 Berlin: Springer

[B2] Cornett DS Reyzer ML Chaurand P Caprioli RM MALDI imaging mass spectrometry: molecular snapshots of biochemical systems Nat Methods 2007 4 10 828 833 10.1038/nmeth1094 17901873

[B3] Schermelleh L Heintzmann R Leonhardt H A guide to super-resolution fluorescence microscopy J Cell Biol 2010 190 2 165 175 10.1083/jcb.201002018 20643879 PMC2918923

[B4] Peng H Bioimage informatics: a new area of engineering biology Bioinf (Oxford, England) 2008 24 17 1827 1836 10.1093/bioinformatics/btn346 PMC2519164 18603566

[B5] Eliceiri KW Berthold MR Goldberg IG Ibáñez L Manjunath BS Martone ME Murphy RF Peng H Plant AL Roysam B Stuurmann N Swedlow JR Tomancak P Carpenter AE Biological imaging software tools Nat Methods 2012 9 7 697 710 10.1038/nmeth.2084 22743775 PMC3659807

[B6] Grueber WB Yang C-H Ye B Jan Y-N The development of neuronal morphology in insects Curr Biol: CB 2005 15 17 R730 R738 10.1016/j.cub.2005.08.023 16139206

[B7] Schneider CA Rasband WS Eliceiri KW NIH Image to ImageJ: 25 years of image analysis Nat Methods 2012 9 7 671 675 10.1038/nmeth.2089 22930834 PMC5554542

[B8] Peng H Ruan Z Long F Simpson JH Myers EW V3D enables real-time 3D visualization and quantitative analysis of large-scale biological image data sets Nat Biotechnol 2010 28 4 348 353 10.1038/nbt.1612 20231818 PMC2857929

[B9] Carpenter AE Jones TR Lamprecht MR Clarke C Kang IH Friman O Guertin DA Chang JH Lindquist RA Moffat J Golland P Sabatini DM CellProfiler: image analysis software for identifying and quantifying cell phenotypes Genome Biol 2006 7 10 R100 10.1186/gb-2006-7-10-r100 17076895 PMC1794559

[B10] Luisi J Narayanaswamy A Galbreath Z Roysam B The FARSIGHT trace editor: an open source tool for 3-D inspection and efficient pattern analysis aided editing of automated neuronal reconstructions Neuroinformatics 2011 9 2–3 305 315 21487683 10.1007/s12021-011-9115-0

[B11] de Chaumont F Dallongeville S Chenouard N Hervé N Pop S Provoost T Meas-Yedid V Pankajakshan P Lecomte T Le Montagner Y Lagache T Dufour A Olivo-Marin J-C Icy: an open bioimage informatics platform for extended reproducible research Nat Methods 2012 9 7 690 696 10.1038/nmeth.2075 22743774

[B12] Linkert M Rueden CT Allan C Burel J-M Moore W Patterson A Loranger B Moore J Neves C Macdonald D Tarkowska A Sticco C Hill E Rossner M Eliceiri KW Swedlow JR Metadata matters: access to image data in the real world J Cell Biol 2010 189 5 777 782 10.1083/jcb.201004104 20513764 PMC2878938

[B13] Kvilekval K Fedorov D Obara B Singh A Manjunath BS Bisque: a platform for bioimage analysis and management Bioinf (Oxford, England) 2010 26 4 544 552 10.1093/bioinformatics/btp699 20031971

[B14] Shamir L Delaney JD Orlov N Eckley DM Goldberg IG Pattern recognition software and techniques for biological image analysis PLoS Comput Biol 2010 6 11 10.1371/journal.pcbi.1000974 PMC2991255 21124870

[B15] Held M a Schmitz MH Schmitz MH Fischer B Walter T Neumann B Olma MH Peter M Ellenberg J Gerlich DW CellCognition: time-resolved phenotype annotation in high-throughput live cell imaging Nat Methods 2010 7 9 747 754 10.1038/nmeth.1486 20693996

[B16] Zhou J Peng H Automatic recognition and annotation of gene expressions of fly embryos Bioinformatics 2007 23 5 589 596 10.1093/bioinformatics/btl680 17237064

[B17] Zhou J Peng H Counting cells in 3D confocal images based on discriminative models 2011 Chicago, IL: ACM Conference on Bioinformatics, Computational Biology and Biomedicine (ACM BCB)

[B18] Velliste M Murphy RF Automated determination of protein subcellular locations from 3D fluorescence microscope images 2002 Washington D.C: Proceedings of the IEEE International Symposium on Biomedical Imaging 867 870

[B19] Jones T Carpenter A Lamprecht M Moffat J Silver S Grenier J Castoreno A Eggert U Root D Golland P SD Scoring diverse cellular morphologies in image-based screens with iterative feedback and machine learning Proc Nat Acad Sci 2009 106 1826 1831 10.1073/pnas.0808843106 19188593 PMC2634799

[B20] Kutsuna N Higaki T Matsunaga S Otsuki T Yamaguchi M Fujii H Hasezawa S Active learning framework with iterative clustering for bioimage classification Nat Commun 2012 3 1032 22929789 10.1038/ncomms2030 PMC3432472

[B21] Shamir L Orlov N Eckley DM Macura T Johnston J Goldberg IG Wndchrm – an open source utility for biological image analysis Source Code Biol Med 2008 3 1 13 10.1186/1751-0473-3-13 18611266 PMC2478650

[B22] Kreshuk A Straehle CN Sommer C Koethe U Cantoni M Knott G Hamprecht FA Automated detection and segmentation of synaptic contacts in nearly isotropic serial electron microscopy images PloS One 2011 6 10 e24899 10.1371/journal.pone.0024899 22031814 PMC3198725

[B23] Theodoridi S Koutroumbas K Pattern Recognition 2009 4 USA, UK: Academic Press

[B24] Hall M Frank E Holmes G Pfahringer B Reutemann P Witten IH The WEKA data mining software: an update SIGKDD Explor 2009 11 1

[B25] Puniyani K Faloutsos C Xing EP SPEX2: automated concise extraction of spatial gene expression patterns from fly embryo ISH images Bioinf (Oxford, England) 2010 26 12 i47 i56 10.1093/bioinformatics/btq172 PMC2881357 20529936

[B26] Ji S Sun L Jin R Kumar S Ye J Automated annotation of Drosophila gene expression patterns using a controlled vocabulary Bioinformatics 2008 24 17 1881 1888 10.1093/bioinformatics/btn347 18632750 PMC2519157

[B27] Carpenter AE Kamentsky L Eliceiri KW A call for bioimaging software usability Nat Methods 2012 9 7 666 670 10.1038/nmeth.2073 22743771 PMC3641581

[B28] Hu MK Visual pattern recognition by moment invariants IRE Trans Info Theory 1962 8 179 187

[B29] Teh C Chin RT On image analysis by the method of moments IEEE Trans Pattern Anal Mach Intell 1988 10 4 496 513 10.1109/34.3913

[B30] Meijering E Jacob M Sarria J-CF Steiner P Hirling H Unser M Design and validation of a tool for neurite tracing and analysis in fluorescence microscopy images Cytometry Part A : J Int Soc Anal Cytol 2004 58 2 167 176 10.1002/cyto.a.20022 15057970

[B31] Chang C-C Lin C-J LIBSVM: a library for support vector machines ACM Trans Intell Syst Technol 2011 2 3 1 27

[B32] Mallat S A Wavelet Tour of Signal Processing 1999 San Diego, CA: Academic

[B33] Icy Spot Detector http://icy.bioimageanalysis.org/plugin/Spot_Detector

[B34] Shamir L Assessing the efficacy of low-level image content descriptors for computer-based fluorescence microscopy image analysis J Microsc 2011 243 3 284 292 10.1111/j.1365-2818.2011.03502.x 21605118

[B35] Chen X Velliste M Weinstein S Jarvik JW Murphy RF Location proteomics – Building subcellular location trees from high resolution 3D fluoresence microscope images of randomly-tagged proteins 2003 San Jose, CA: SPIE 296 306

[B36] Lu J Neuronal tracing for connectomic studies Neuroinformatics 2011 9 2–3 159 166 21340747 10.1007/s12021-011-9101-6

[B37] Grueber WB Yang CH Ye B Jan YN The development of neuronal morphology in insects Curr Biol 2005 15 730 738 10.1016/j.cub.2005.08.023 16139206

[B38] Trainable Segmentation Plugin http://fiji.sc/Trainable_Segmentation_Plugin

[B39] Peng HC Long FH Zhou J Leung G Eisen M Myers E Automatic image analysis for gene expression patterns of fly embryos BMC Cell Biol 2007 8 s7 10.1186/1471-2121-8-S1-S7 17634097 PMC1924512

