# Supplementary material for: BIOCAT: a pattern recognition platform for customizable biological image classification and annotation
Source: BMC Bioinformatics. 2013 Oct 4;14:291. doi: 10.1186/1471-2105-14-291 (PMC3854450; doi:10.1186/1471-2105-14-291)
Supplement: Additional file 1: Table S1 — Biological image sets. [file 1471-2105-14-291-S1.doc]

**Additional file 1: Table S1 Biological image sets**

| **Image set** | **Image example** | **Image type** | **Image size (Number of Pixels)** | **# of images** | **#of classes** |
| --- | --- | --- | --- | --- | --- |
| K1502D | 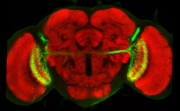 | Fluorescence | 50K (530*998 24 bit) | 12 training 8 testing | 4 |
| K1503D | 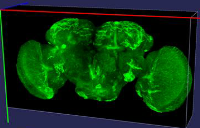 | Fluorescence | 83M (512*512*108 24 bit) | 20 | 4 |
| CHO | 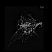 | Fluorescence | 190K (512*382) | 340 | 5 |
| Binucleate | 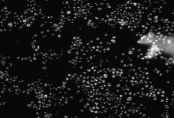 | Fluorescence | 2.5M (1280*1024 16 bit ) | 40 | 2 |
| LiverGenderCR | 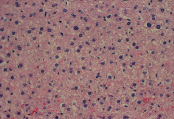 | Brightfield | ~300K (1388*1040 32 bit) | 303 | 2 |
| LiverGenderAL | 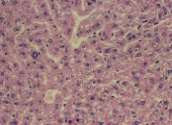 | Brightfield | 2.8M (1388x1040 32 bit) | 522 | 2 |
| MuscleAge | 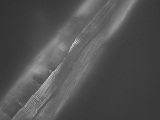 | Fluorescence | 2.8M (1600x1200 16 bit) | 252 | 4 |
| Pollen | 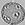 | Phase Contrast | 225 (25*25) | 630 | 7 |
| Hela | 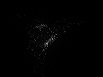 | Fluorescence | 150K(382x382 16 bit) | 860 | 10 |
| Termbulb | 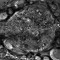 | DIC | 177K (300x300 16 bit) | 970 | 7 |
| Lymphoma | 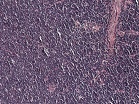 | Brightfield | 4M (1388x1040 32 bit) | 375 | 3 |
| LiverAging | 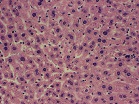 | Brightfield | 2.8M (1388x1040 32 bit) | 850 | 4 |
| RNAi | 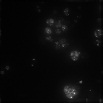 | Fluorescence | 2M (1024x1024 16 bit) | 200 | 10 |

Sets are from http://ome.grc.nia.nih.gov/iicbu2008/ [21] or BIOCAT.
