# Supplementary material for: BIOCAT: a pattern recognition platform for customizable biological image classification and annotation
Source: BMC Bioinformatics. 2013 Oct 4;14:291. doi: 10.1186/1471-2105-14-291 (PMC3854450; doi:10.1186/1471-2105-14-291)
Supplement: Additional file 2: Table S2 — Algorithm chain comparison algorithm chains for K150 3D. [file 1471-2105-14-291-S2.doc]

**Additional file 2: Table S2** **Algorithm chain comparison algorithm chains for K150 3D**

| **Chain** | **Anisotropic wavelet** | **Hu moments** | **Fisher selector** | **SVM** | **RF** | **NN** | **Accuracy (%)** | 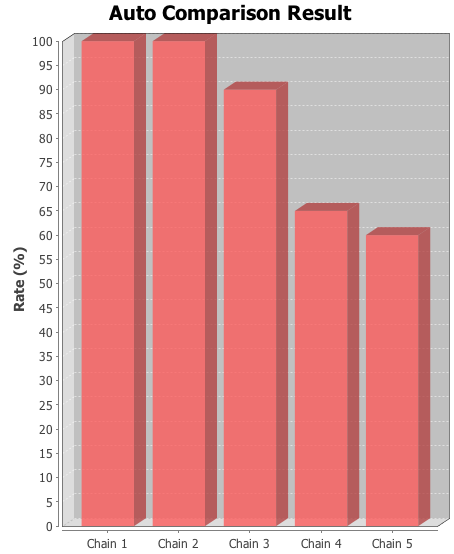 |
| --- | --- | --- | --- | --- | --- | --- | --- | --- |
| 1 | Y | N |  | Y |  |  | 100 |
| 2 | Y | N | Y | Y |  |  | 100 |
| 3 | Y | N | Y |  | Y |  | 90 |
| 4 | N | Y |  |  | Y |  | 65 |
| 5 | N | Y |  |  |  | Y | 60 |
