# Supplementary material for: BIOCAT: a pattern recognition platform for customizable biological image classification and annotation
Source: BMC Bioinformatics. 2013 Oct 4;14:291. doi: 10.1186/1471-2105-14-291 (PMC3854450; doi:10.1186/1471-2105-14-291)
Supplement: Additional file 3: Table S3 — Algorithm chain comparison for classification of 3D ROI around a fruit fly nuclei center). [file 1471-2105-14-291-S3.doc]

**Additional file 3: Table S3** **Algorithm chain comparison for classification of 3D ROI around a fruit fly nuclei center**

| **Chain** | **3D anisotropic wavelet** | **3D moments** | **SVM** | **Random-forest** | **ROI recognition rate (%)** | 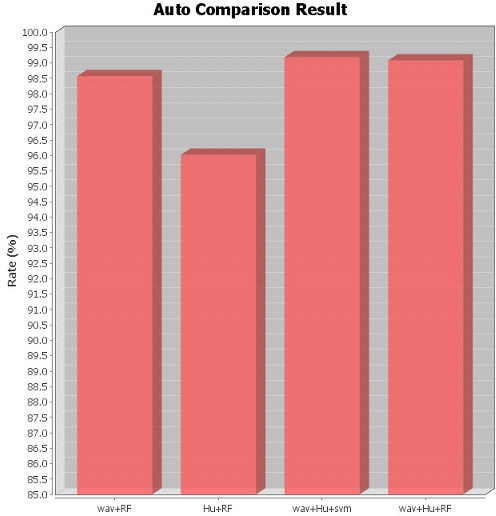 |
| --- | --- | --- | --- | --- | --- | --- |
| 1 | Y | N |  | Y | 98.6 |
| 2 | N | Y |  | Y | 96.0 |
| 3 | Y | Y | Y |  | 99.2 |
| 4 | Y | Y |  | Y | 99.1 |
